# Analysis and Compensation of Bias Drift for a Micromachined Spinning-Rotor Gyroscope with Electrostatic Suspension

**DOI:** 10.3390/s20061799

**Published:** 2020-03-24

**Authors:** Shunyue Wang, Fengtian Han

**Affiliations:** Department of Precision Instrument, Tsinghua University, Beijing 100084, China; wsy15@mails.tsinghua.edu.cn

**Keywords:** micromachined electrostatically suspended gyroscope, temperature-induced drift, bias drift compensation, time drift, bias stability

## Abstract

Bias stability is one of primary characteristics of precise gyroscopes for inertial navigation. Analysis of various sources of the bias drift in a micromachined electrostatically suspended gyroscope (MESG) indicates that the bias stability is dominated by the temperature-induced drift. The analytical results of temperature drift resulting from the rotor structure and capacitive position sensing electronics are modeled and analyzed to characterize the drift mechanism of the MESG. The experimental results indicate that the bias drift is mainly composed of two components, i.e., rapidly changing temperature drift and slowly changing time drift. Both the short-term and long-term bias drift of the MESG are tested and discussed to achieve online bias compensation. Finally, a neural network based-bias compensation scheme is presented and verified experimentally with improved bias stability of the MESG.

## 1. Introduction

Micromachined gyroscopes have attracted much attention in inertial measurements and navigation applications wherein lightweight, low-cost and high-performance sensors are crucial [1,2]. In order to minimize the mechanical friction and wear of micromachined spinning-rotor gyroscopes, various suspension schemes, including contactless gas-lubricated bearings [3], liquid-suspended bearings [4,5,6] and electrostatically and electromagnetically suspended bearings [7,8,9] have been studied and fabricated for better performance and longer lifetime. Among them, the micromachined electrostatically suspended gyroscope (MESG) is comparatively compatible with the existing micro-electromechanical system (MEMS) fabrication process [10,11]. Most of the published work in the literature focused on design [12,13,14], fabrication [11,15], suspension [7] and rotation [16] of the MESG. In this paper, the temperature characteristics of the MESG bias are studied first. Currently, most of these MESG devices are fabricated with glass/silicon/glass triple-wafer bonding and suspended electrostatically in five degrees of freedom (DOFs). Temperature-induced drift is one of dominant error sources for the MESG with its spinning rotor suspended by a frictionless electrostatic bearing, as the gyroscope output is sensitive to environmental temperature variation resulting from thermal impact on interface electronics and sensing structure [17].

The bias stability of gyroscopes is the most crucial performance gauge for inertial navigation applications [18]. Ambient temperature variation inevitably results in bias drift and performance degradation [19,20]. Constant temperature control and temperature drift compensation are the two usual approaches to reducing greatly the bias drift and enhancing the gyroscope performance. Generally, ultra-high precision gyroscopes are typically operated in a constant temperature but bulky chamber to provide ultra-low bias drift [20,21,22]. By contrast, most of gyroscopes generally operate with temperature-based model compensation to attenuate the bias drift and thus enhance the gyroscope stability, which is an effective and inexpensive solution. A support vector machine (SVM) method was used to compensate the temperature drift of a dynamically tuned gyroscope, which reduces the average drift down to 0.003°/h from a 735 daily averaged temperature drift data and real-time temperature data [17]. Recently, the modeling and compensation of temperature-induced drift have also been studied for high-performance MEMS gyroscopes. A drift self-compensation scheme for silicon quadruple mass gyroscopes was reported by using its resonant frequency measurement as an embedded thermometer, rather than placing a temperature sensor in close proximity to the gyroscope chip [23]. A back-propagation (BP) artificial neural network was used to compensate the bias drift of MEMS gyroscopes, which exhibits a non-linear model between the gyroscope output and ambient temperature [24]. The bias drift of a quadruple mass vibratory gyroscope was compensated by the mode-matching method, and the bias stability reaches as low as 0.2°/h for a time duration of half a month [25]. However, the temperature drift compensation and the evaluation of the existing MEMS vibratory gyroscopes are mostly for short-time operation. Heretofore, very little research has been reported on long-term temperature drift characteristics of micromachined spinning-rotor gyroscopes, which have the potential to be used for high precision and long-term inertial measurements. Tokyo university has reported the static MESG data for 85 h with a sampling time of 2.2 ms, but only the most stable 4-h data are used to analysis the bias instability by Allan variance [26]. To achieve high performance of the MESG for long-term and wide-temperature operation, it is significant to analyze the mechanism and characteristics of long-term temperature drift to improve the design of MESG sensing structure and associated electronics. In addition, specific compensation methods can be utilized to suppress the temperature drift greatly and improve the bias stability of MESGs as well.

This purpose of this paper is to examine the temperature-induced bias drift for a prototype MESG with a high-speed spinning rotor. The theoretical analysis and experimental evaluation of the thermal rotor deformation and sensing electronics drift are presented to characterize the temperature-induced bias drift of MESGs. The MESG measurements for up to 70 h in a temperature test chamber are presented and discussed to examine the long-term bias drift, which comprises rapidly changing temperature drift and slowly changing time drift. A BP neural network-based drift compensation scheme is experimentally validated to offer much improved long-term bias stability for a duration up to 70 h.

## 2. Device Description

### 2.1. Device Structure

**Figure 1:**
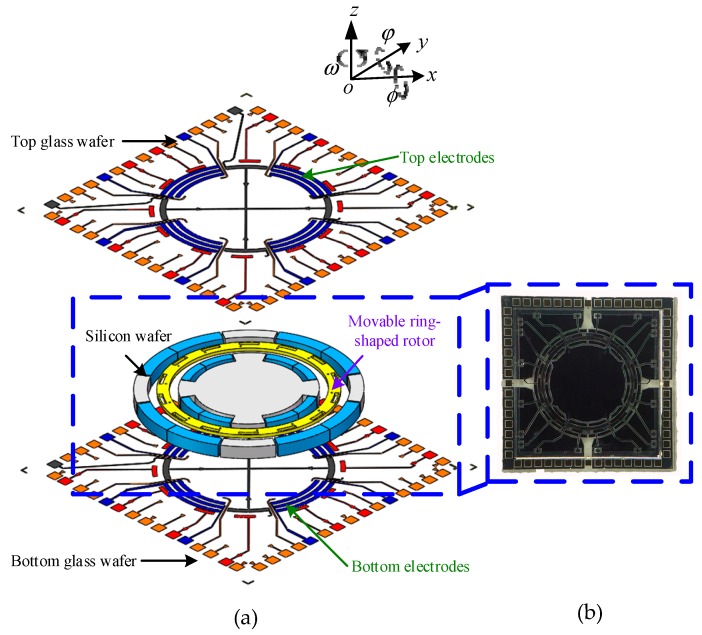
Micromachined electrostatically suspended gyroscope: (**a**) an exploded view of the device and (**b**) the fabricated die.

The MESG presented here consists of a glass-silicon-glass triple-wafer bonding structure with a complex electrode pattern, as shown in the exploded view and a fabricated die of the MESG in Figure 1. A typical structure of the MESG consists of two Pyrex 7740 glass wafers, and one contactless ring-shaped rotor which is formed in the intermediate silicon wafer [8]. The sensing and actuation electrodes around the rotor are used to suspend the rotor electrostatically in DOFs and spin up the rotor at high speed so as to function as a two-DOF angular rate gyroscope. Planar electrodes symmetrically sputtered on the top and bottom glass wafers are used for axial suspension of the rotor in three DOFs: translation along the *z*-axis and rotation around the two in-plane axes. Moreover, the outmost electrodes on each glass wafer are used for rotation drive of the rotor which is operated as a planar variable-capacitance micro-motor. The silicon wafer is etched to form the ring-shaped rotor and truncated pie-shaped radial electrodes which are used for radial suspension of the rotor along the *x*- and *y*-axes. Main design parameters of the MESG structure are listed in Table 1. 

The MEMS device was fabricated using bulk microfabrication based on silicon on glass (SOG) technique, which includes several key processes, such as high-aspect-ratio dry etching, twice glass-silicon anodic bonding, aluminum sacrificial layer removal and vacuum packaging. The size of each fabricated die is 6.5 × 6.5 × 1.1 mm, as depicted in Figure 1b [27]. 

### 2.2. Scale Factor and Full-Scale Range

Basically, the MESG is a dual-axis angular rate sensor when the rotor is spinning at a constant and high speed. If no torque is applied on the rotor, it will maintain its orientation in the inertial space. However, if an angular rotation orthogonal to the spin axis occurs, a precession torque generated by the rebalance loop will force the rotor to its null position. The scale factor is one of the main design parameters for MESGs, which describes the response of the gyroscope output *V*_Ω_ under an angular-rate input Ω_x._ The precession of the spinning rotor is governed by the equation:

(1) $$\left|H\Omega_{x}\right|=V_{\Omega }K_{v},$$

where *H* = *J*_z_·*ɷ*_0_ is the angular momentum of the rotor; *K*_v_ is the electrostatic torquer gain of the rebalance loop; *ɷ*_0_ and *J*_z_ are the rated spin rate and moment of inertia of the rotor spinning around the *z*-axis, respectively.

The scale factor *K*_f_ in V/(° s^−1^) can be expressed by

(2) $$K_{f}=\frac{V_{\Omega }}{\Omega_{x}}=\frac{J_{z}\cdot \omega_{0}}{K_{v}}.$$

Equation (2) indicates that the scale factor of MESGs is proportional to the rated spin rate ω_0_. Note that the scale factor stability is also closely related with the constant speed accuracy of the rotation control loop. To obtain high sensitivity and excellent stability, it is essential to have a high and precise spin rate of the rotor during design of the MESG structure, rotation electronics and vacuum packaging [8]. 

In principle, the full-scale range of the MESG in °/s can be given by

(3) $$\Omega_{r\_\max}=\frac{V_{\mathrm{br}}}{K_{f}},$$

where *V*_br_ is the bias voltage applied on the axial electrodes, which is also the allowable maximum voltage of the MESG output. According to Equation (2), the scale factor reaches 26.1 mV/(°/s) at the rated spin rate of 1 × 10^4^ rpm. When the bias voltage is set at 7.2 V, the full-scale measurement range of the MESG is ± 275.86 °/s. It is clear that the scale factor increases as the spin rate rises while the full-scale range will decrease as the scale factor becomes larger.

### 2.3. Vacuum Packaging 

As a spinning-rotor gyroscope, a vacuum environment is essential for operation of the MESG to reduce viscous drag dramatically and thus to enable ultra-high rotation of the rotor. High vacuum level as low as 0.01 Pa can minimize the effect of residual air-film damping and achieve a desirable spin rate over 1 × 10^4^ rpm with a rotation voltage below 7.6 V, which is essential to dramatically enhance the overall performance of the MESG.

**Figure 2:**
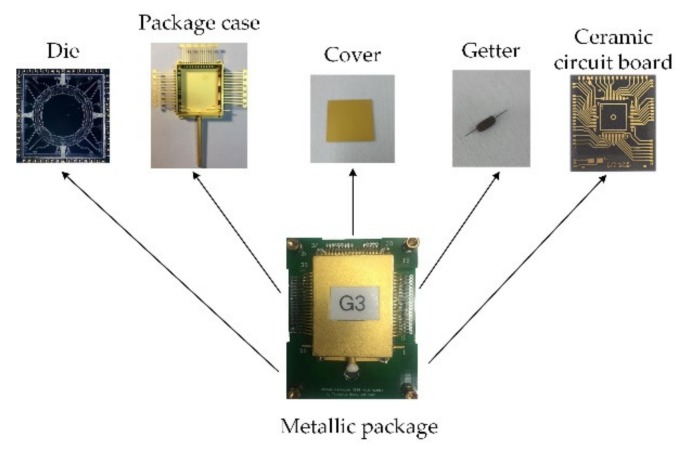
Schematic diagram of the device-level vacuum packaging of the MESG.

Figure 2 shows a schematic illustration of the device-level vacuum packaging using a metallic chip package. To reduce the number of the package pins, a ceramic board was used to rearrange and combine part of the die’s pins, which have the same electrical function. Firstly, the 60-pin die was fixed to the ceramic circuit board using JM7000 adhesive (Henkel, Dusseldorf, Germany) baking at 175 °C for 2 h. Secondly, the MESG die was wire-bonded to the 55-pin ceramic circuit board. Then the ceramic board was further wire-bonded to the 55-pin metallic chip package. Thirdly, the parallel seam welding technique was used to seal the metallic package with the cover. When the two conical electrodes roll along the edge of the metal cover at a speed of 50 mm/s, a 1 kHz power pulse signal with a pulse width of 3 ms and a peak power of 2500 W was applied on the two electrodes to generate Joule heat. Then a partial molten state was formed between the cover and the package frame, and the sealing process was completed after solidification of the soldering seam. Fourthly, both the ceramic board and metallic package were high-temperature degassed in a vacuum chamber to bake out the water and gas that adhered in the surface. In order to minimize gas evolution from the material of die, the ceramic board and metallic package, a 160 h, 10^−5^ Pa and 130 °C baking-out procedure was used to drive out chemically dissolved water, carbon dioxide, nitrogen and oxygen from the glass and hydrogen from the metal. Finally, a heating current of 3.3 A was applied for 10 min to activate the getter and thereafter maintain high vacuum of the MESG packaging.

It is essential for the vacuum packaging to provide a required initial vacuum as well as desirable duration in years after sealing [28]. The lifetime of device-level encapsulation is a key measure with which to evaluate the reliability of most sensor products in vacuum [29,30]. Any defects during the sealing and packaging process could cause failure of MESGs or performance degradation over time. Therefore, it is important to characterize the vacuum maintenance duration of the MESG which could provide valuable information for lifetime evaluation of the MESG.

The relationship between the viscous damping effect and speed decay duration of the rotor has been experimentally studied for MESGs [16]. When the rotation drive torque was removed, the rotation speed would decrease due to weak damping effect of the residual gas inside the vacuum package. Therefore, the speed decay time is a measure of the real vacuum pressure inside the package. Define the decay time constant as *τ* = *J_z_* /*b* where *b* is the viscous damping coefficient associated with the vacuum pressure; then the instantaneous rotor speed *ω* (*t*) during the decay process can be expressed as

(4) $$\omega (t)=\omega (0)\times e^{-\frac{t}{\tau }},t\geq 0.$$

**Figure 3:**
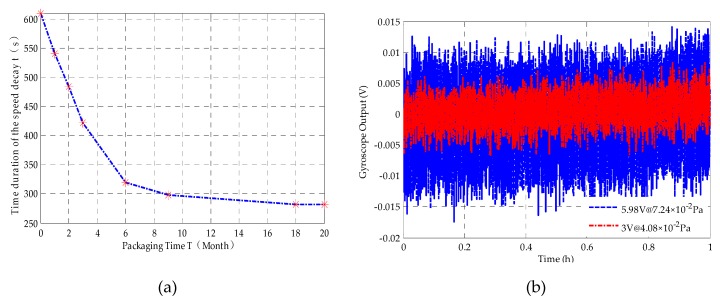
(**a**) Stability of vacuum tests up to 20 months and (**b**) gyroscope noise at different drive voltages.

So the gas pressure inside the vacuum package can be estimated by *τ*, which can be obtained from speed decay experiments. The stability of vacuum maintenance in one MESG has been monitored by measuring periodically the speed decay time (*τ*) up to 20 months. The experimental results show that the MESG device works stably during 20 months and the estimated vacuum pressure is still below 0.1 Pa. As shown in Figure 3a, the speed decay duration decreases rapidly in the first six months and stays virtually constant after 18 months. Figure 3a indicates that the speed decay duration was reduced from 610 s to 282 s after a 20-month encapsulation. 

Equation (2) indicates that high spin rate would contribute to enhancing the scale factor of the gyroscope. The higher the vacuum level in the device package, the higher the spin rate that could be reached. Figure 3b shows that at the same spin rate of 1.0 × 10^4^ rpm, high vacuum level will contribute to low rotation drive voltage and thus reduce gyroscope output noise.

The gyroscope output noise is induced by a combination of the electrostatic suspension voltage and the rotation drive voltage. Note that the rotation drive torque is proportional to the square of the rotation voltage and the precession control torque varies linearly with the MESG output voltage. Then, by assuming these two noise sources are independent, the overall noise contributions to the gyroscope’s output from these two sources can be approximated by

(5) $$V_{n}=\sqrt{V_{\mathrm{ns}}^{2}+{\left(k_{n}V_{r}^{2}\right)}^{2}},$$

where *V*_n_ and *V*_ns_ are the gyroscopic output noise and suspension voltage induced noise, *V*_r_ and *k*_n_ are the rotation drive voltage and its noise coefficient, respectively. The experimental results in Figure 3b show that the root-mean-square values of *V*_ns_ and *k*_n_ are 1.8 × 10^−3^ V and 1.95×10^−4^ by data fitting in Equation (5), respectively. It is clear that the gyroscope output noise can be reduced greatly with higher vacuum to permit low rotation drive voltage. Equation (5) indicates that the gyroscopic noise will fall by 67% as the rotation voltage reduces from 5.98 V to 2.85 V. Therefore, a high vacuum packaging better than 4 × 10^−2^ Pa is the first prerequisite to realizing low-noise MESGs operating at the spin rate of 1.0 × 10^4^ rpm.

## 3. Temperature Dependent Characteristics of the MESG

Theoretically, the variation of environment temperature and the heat generated by associated electronics of MESGs will lead to the change of the temperature field distribution, which is the dominant source of the temperature drift. The temperature-dependent output drift of the MESG could result from thermal variations in both the device structure and electronics.

### 3.1. Rotor Deformation-Induced Bias Drift

#### 3.1.1. Theoretical Analysis of the Rotor Thermal’s Deformation

The temperature drift of the gyroscope output comes from both the average temperature variation and internal temperature gradient fluctuation. The non-uniformly distributed or changing temperature field on the rotor could result in the variation of the angular momentum and the deviation of the center of mass from the center of support. Here, the temperature drift caused by thermal deformation of the rotor structure is evaluated approximately by an analytical model.

Rotor deformation can be described by quasi-static thermoelastic theory, as the MESG works under ordinary heat exchange conditions. The displacement and temperature field are described by a simplified thermoelastic motion equation.

(6) $$(1-2v^{*})\nabla^{2}U+graddivU-2(1+v^{*})\alpha_{T}gradT=0,$$

where $v^{*}$ is the poisson’s ratio; U is displacement vector; α_T_ is the coefficient of thermal expansion; *T* is the temperature; and $\nabla^{2}$ is the Laplace operator. 

**Figure 4:**
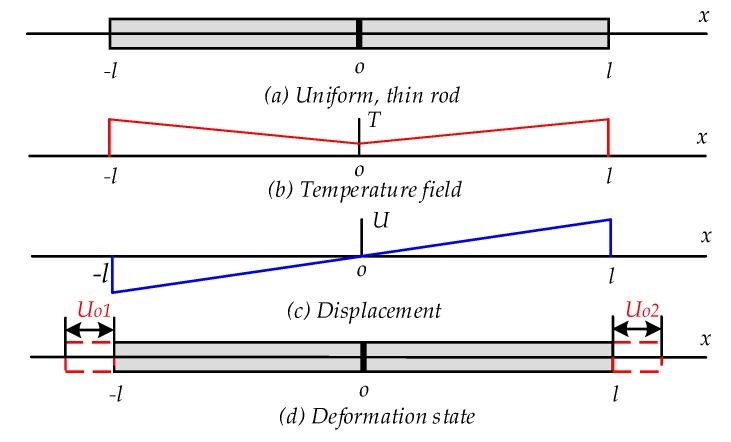
A schematically thermoelastic deformation of the MESG rotor.

A uniform, thin rod is assumed to represent the ideal rotor suspended electrostatically, as shown in Figure 4.

Assuming that the temperature field varies along the rotor in accordance with linear temperature variation, then the expression of the temperature variation is

(7) $$T=\frac{T_{0}-25}{l}x+25,$$

where *l* is the rotor diameter, *T*_0_ is the environmental temperature and *x* is the rotor deformation. In a one-dimensional coordinate system, the expression of the thermoelastic equation is simplified as

(8) $$\frac{dU(x)}{dx}=\frac{1+v^{*}}{1-v^{*}}\alpha_{T}T(x).$$

Equation (9) gives the solution of Equation (8) with the boundary condition *U*(0) = 0.

(9) $$U(x)=\frac{1+v^{*}}{1-v^{*}}\alpha_{T}[\frac{T_{0}-25}{2l}x^{2}+25x].$$

**Figure 5:**
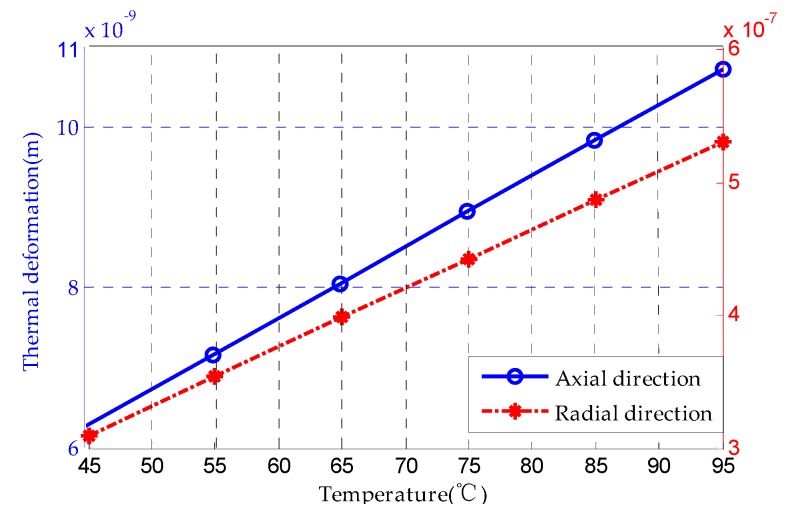
Theoretical results of rotor thermal deformation along axial and radial directions.

Figure 5 shows the analytical results of the rotor thermal deformation along the axial and radial axes. It is indicated that the coefficients of thermal deformation along the axial and radial directions are 8.964 × 10^−11^ m/°C and 4.426 × 10^−9^ m/°C, respectively. 

#### 3.1.2. Bias Drift Caused by Thermal Expansion of the Rotor Structure

**Figure 6:**
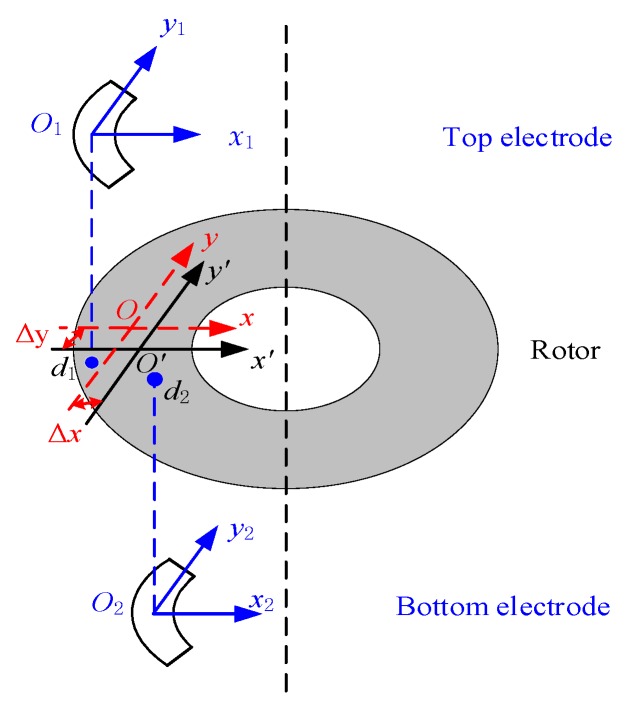
Alignment error caused by thermal expansion of the rotor.

Ideally, there is no alignment error between the rotor and the top/bottom electrodes. However, the glass-silicon anodic bonding process inevitably leads to alignment error in the order of microns. As shown in Figure 6, there are constant alignment errors between the origins of top electrode *O*_1_*x*_1_*y*_1_*z*_1_ and bottom electrode *O*_2_*x*_2_*y*_2_*z*_2_ when they are projected onto the *O*’*x*’*y*’*z*’ coordinates of the rotor wafer. According to the experience in bonding alignment process, the alignment precision can be controlled within 2–5 μm. The *O*’*x*’*y*’*z*’ coordinates will move to *Oxyz* due to the thermal expansion effect of the rotor. As shown in Figure 6, the origin of the *Ox*_1_*y*_1_*z*_1_ coordinate is projected onto the silicon rotor as *d*_1_= (Δ*x*, Δ*y*, *h*/2)^T^, which denotes in the *Oxyz* coordinates. Similarly, the origin of the *Ox*_2_*y*_2_*z*_2_ coordinate is projected onto the silicon rotor as *d*_2_= (Δ*x*, Δ*y*, −*h*/2)^T^. Therefore, the alignment error variation caused by thermal expansion introduces electrostatic disturbance torque, which affects the angular rate measurements along both the *x* and *y* directions.

In an electrostatically suspended system of the MESG, define the suspension stiffness along the *x*, *y* and *z* axes as [*k*_x_, *k*_y_, *k*_z_]. When the rotor suffers from a vector input acceleration [*a*_x_, *a*_y_, *a*_z_], the origin will deviate to the coordinates (−*ma*_x_/ *k*_x_, −*ma*_y_/ *k*_y_, −*ma*_z_/ *k*_z_). The moment arms *l*_1_ and *l*_2_ formed by the top and bottom electrodes can be expressed as

(10) $$\left\{\begin{array}{l} l_{1}={\left(\Delta x+ma_{x}/k_{x},\Delta y+ma_{y}/k_{y},h/2+ma_{z}/k_{z}\right)}^{T}\\ l_{2}={\left(\Delta x+ma_{x}/k_{x},\Delta y+ma_{y}/k_{y},-h/2+ma_{z}/k_{z}\right)}^{T} \end{array}\right..$$

When a small offset *d*’ due to thermal expansion occurs along the *z* axis of the frame, the electrostatic forces generated by the top and bottom electrodes on the rotor are as

(11) $$\left\{\begin{array}{l} F_{1}=(0,0,-\frac{\varepsilon \alpha (R_{0}{}^{2}-R_{i}{}^{2})\cdot {\left(V_{bz}-V_{fz}\right)}^{2}}{{\left(d_{z}-d^{\prime }\right)}^{2}}{)}^{T}\\ F_{2}=(0,0,-\frac{\varepsilon \alpha (R_{0}{}^{2}-R_{i}{}^{2})\cdot {\left(V_{bz}+V_{fz}\right)}^{2}}{{\left(d_{z}+d^{\prime }\right)}^{2}}{)}^{T} \end{array}\right.,$$

where *α* = π/3 is the arc angle of the axial electrodes; *ε* is the permittivity; *V*_bz_ and *V*_fz_ are the bias voltage and feedback voltage of the *z*-axis, respectively. The electrostatic moment caused by the alignment error is

(12) $$M_{f}=l_{1}\times F_{1}+l_{2}\times F_{2}=\frac{2\varepsilon \alpha (R_{0}{}^{2}-R_{i}{}^{2})\cdot V_{bz}{}^{2}}{d_{z}{}^{2}}{\left[\Delta y,\Delta x,0\right]}^{T}.$$

The thermal expansion-induced moment produces an angular rate response ${\dot{\varphi }}_{x}$ of the MESG along the *x* axis is

(13) $${\dot{\varphi }}_{x}=\frac{H\times M_{x}}{H^{2}}.$$

According to the analytical results of thermal deformation in Section 3.1.1, we know that Δ*x/dT* = Δ*y/dT* = 4.43 × 10^−9^ m/°C. Then, the bias temperature drift of the MESG resulted from the rotor thermal expansion is 3.59 × 10^−3^ mV/°C based on Equation (13).

### 3.2. Radiative Thermal Transfer Model of the MESG

As the rotor is suspended in high vacuum without any mechanical contact, the temperature stabilization or change on the rotor is mainly caused by radiative thermal transfer rather than solid thermal conduction. Therefore, it is necessary to calculate the thermal equilibrium time of the rotor under radiative thermal transfer only. In order to simplify the analysis, the thermal transfer process from the environment to the electrode chamber is omitted. Here we only discuss the most critical thermal transfer response between the electrode cavity and the contactless silicon rotor.

Based on the energy balance relationship of microelement in the heat transfer theory, the governing equation for the MESG can be written as follows.

(14) $$-\nabla \cdot q(r,t)+g(r,t)=\rho C_{p}\frac{\partial T(r,t)}{\partial t},$$

where ***T*** is the temperature inside the microelement; *C*_p_ is the specific heat of the material; *g* is the heat energy generation rate per unit volume; ***q*** is the heat flux conducting along the boundary normal direction; ∇·q is the heat flux gradient conducting along the boundary normal direction; *ρ* is the material density; and ***r*** is the distance vector.

Calculating the volume integral of the heat equilibrium equation (Equation (14)) yields

(15) $$-\int_{V}\nabla \cdot q(r,t)dV+\int_{V}g(t)dV=\int_{V}\rho C_{p}\frac{\partial T(t)}{\partial t}dV.$$

The Gaussian integral formula is used on the first term on the left side of Equation (15), and the radiation law is used on the second term on the left side. Then Equation (15) can be rewritten as:

(16) $$\tau \frac{\partial T(t)}{\partial t}+T(t)=T_{\infty }+\frac{\tau }{\rho C_{p}}\Delta g(t)+\Delta T_{a}(t).$$

The solution of Equation (16) can be expressed as

(17) $$T(t)=T_{\infty }+(T_{0}-T_{\infty })e^{-t/\tau }+\frac{1}{\tau }\int_{0}^{t}\left(\frac{\tau }{\rho C_{p}}\Delta g(\upsilon )+\Delta T_{a}(\upsilon \right))e^{-(t-\upsilon )/\tau }dv,$$

where $\tau =\frac{\rho C_{p}\pi (r_{o}{}^{2}-r_{i}{}^{2})\cdot h}{[2\pi r_{0}h+2\pi (r_{o}{}^{2}-r_{i}{}^{2})]\cdot \varepsilon_{1}\delta T_{a}{}^{3}}$ and $T_{\infty }=T_{a0}+\frac{\tau }{\rho C_{p}}g_{0}$. Then Equation (18) is obtained based on Fourier series $\Delta g(t)=\sum_{n=-\infty }^{\infty }a_{n}e^{-jn\omega t}$ and $\Delta T_{a}(t)=\sum_{n=-\infty }^{\infty }b_{n}e^{-jn\omega t}$

(18) $$T(t)=T_{\infty }+(T_{0}-T_{\infty })e^{-t/\tau }+\sum_{n=-\infty }^{\infty }(\frac{\tau }{\rho C_{p}}a_{n}+b_{n})\frac{1}{1+j\tau n\omega }(e^{jn\omega t}-e^{-t/\tau }).$$

The steady-state rotor temperature response can be obtained when t→∞

(19) $$\underset{t\to \infty }{\lim}T(t)=T_{\infty }+\sum_{n=-\infty }^{\infty }(\frac{\tau }{\rho C_{p}}a_{n}+b_{n})\frac{1}{1+j\tau n\omega }e^{jn\omega t}.$$

As can be seen from Equations (18) and (19), the rotor temperature response changing from an initial state to its equilibrium is in an exponential decay process with a time constant *τ* = 711.5 s, when the silicon emissivity is *ε*_1_ = 0.05 and *T*_a_ is 300 K. Here *δ* = 5.67 × 10^−8^ *W*/(m^2^·K^4^) is the blackbody radiation constant coefficient. The thermal equilibrium time of the suspended rotor is calculated as 3*τ* = 35.6 min, which roughly governs the temperature stabilization process of the MESG device.

### 3.3. Temperature Drift Analysis of the Position Sensing Circuit

**Figure 7:**
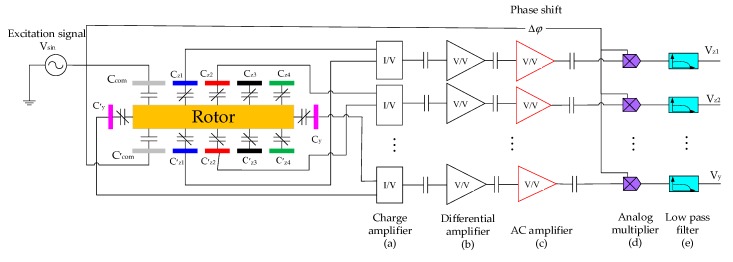
A schematic of the rotor displacement sensing circuit.

Six capacitive position sensing channels, as illustrated in Figure 7, are used to detect the rotor position and thus realize electrostatic suspension of the rotor in five DOFs. A sinusoidal excitation signal with an amplitude of 1.48 V and frequency of 1 MHz is applied to the common electrode C_com_. When the rotor deviates from the equilibrium position, a differential capacitance change is generated between the rotor and associate sensing electrodes; i.e., Δ*C*_i_ =*C*_i_-*C’*_i_ (i=*z*1, *z*2, *z*3, *z*4, *x*, *y*). The unbalanced current is then converted to the sensing voltage by a pair of differential charge amplifiers with very high input impedance. The ensuing AC amplifier stage functions as a band-pass filter with a gain of up to 150. The displacement sensing signal from the AC amplifier is a high frequency amplitude modulation wave and is fed to an analog multiplier for phase sensitive modulation. Finally, the position sensing output voltage is obtained by low-pass filtering at a cut-off frequency of 20 kHz. 

Since the series capacitors are used to couple the AC position sensing signal—see the capacitors shown in Figure 7a–c—the offset drifts of the charge amplifiers, differential amplifiers and AC amplifier caused by ambient temperature variation can be ignored. However, the temperature-induced offset voltage change of the analog multipliers and low-pass filters have to be considered to evaluate the thermal stability of the position sensing outputs. 

According to datasheets of the amplifiers used in the MESG, the temperature drift coefficient of input offset voltage for the low-pass filter is as low as 2 μV/°C. The analog multiplier is the most basic application of AD734AN (Analog Devices Inc., Norwood, MA, USA), where no external components are needed to constitute the multiplier. The temperature drift range of the multiplier output, which is mainly caused by the input offset voltage drift, is estimated from −0.9 mV/°C to 0.42 mV/°C according to AD734AN’s datasheet. 

To test the temperature drift of the capacitance sensing circuit, low temperature-drift capacitor pairs were used to replace the differential rotor-electrode capacitance of the MESG, as depicted in Figure 7. Then the entire sensing circuit was put into a temperature controlled chamber to evaluate the temperature-induced output drift from 40 °C to 60 °C in a step of 10 °C. The above-mentioned experiments were repeated three times to ensure the repeatability of the measured temperature drift coefficient, which yielded −0.2895 mV/°C, −0.2848 mV/°C and −0.2571 mV/°C in three measurements, respectively. The temperature drift coefficient of the sensing circuit has a mean value of −0.2771 mV/°C, which is within the temperature drift range of the analog multiplier. The experimental result agrees well with the predication that the analog multiplier dominates the temperature drift of the position sensing circuit. 

### 3.4. Gyroscopic Output Drift Caused by the Temperature Drift of Position Sensing Circuit

**Figure 8:**
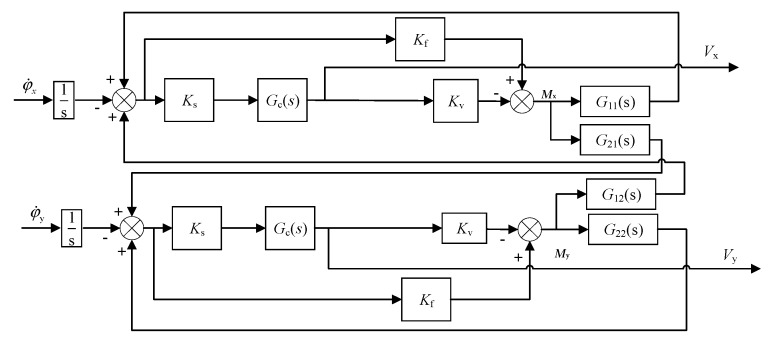
Block diagram of the dual-axis MESG rebalance loop.

A dual-axis rebalance loop for operation and measurement of the two-DOF MESG is shown in Figure 8. When an angular rate input is applied to the gyroscope, the dual-axis torque-rebalance loop stabilizes the rotor over its dynamic range and provides precise measurement signals *V_x_* and *V_y_*, as the Simulink simulation model shows in Figure 8.

In order to design the MESG rebalance loop with desirable performance, two lag-lead compensators are used to provide appropriate phase compensation, as given by Equation (20), which stabilizes the open-loop unstable dual-axis system by feedback.

(20) $$G_{c}(s)=150\frac{\left(s+50)(s+4000\right)}{\left(s+1)(s+40000\right)}.$$

Figure 8 indicates that the dual-axis rebalance loop describes the relationship between the angular rate inputs ${\dot{\varphi }}_{x},{\dot{\varphi }}_{y}$ and the gyroscopic outputs *V_x_*, *V_y_*, where G(s) governs the dynamics of the spinning rotor in the form of

(21) $$G(s)=\left[\begin{array}{cc} G_{11}(s) & G_{12}(s)\\ G_{21}(s) & G_{22}(s) \end{array}\right]=\left[\begin{array}{cc} \frac{J_{e}}{J_{e}{}^{2}s^{2}+H^{2}} & \frac{-H}{s(J_{e}{}^{2}s^{2}+H^{2})}\\ \frac{H}{s(J_{e}{}^{2}s^{2}+H^{2})} & \frac{J_{e}}{J_{e}{}^{2}s^{2}+H^{2}} \end{array}\right];$$

G12(s) and G21(s), and G11(s) and G22(s) describe the precession motion and rigid body characteristics of the spinning-rotor gyro, respectively [14]. Main parameters of the rebalance loop are listed in Table 2.

Simulink simulation results indicate that the temperature-induced bias drift rate of the MESG output is 8.7 mV/°C when the temperature drift coefficient of the capacitive position sensing is set at its measured mean of 0.28 mV/°C. It is clear that the bias drift induced from the position pick-off electronics is virtually four orders of magnitude larger than that from thermal expansion of the rotor structure. 

## 4. Experimental Results and Bias Drift Compensation

### 4.1. Experimental Set-Up for the MESG

**Figure 9:**
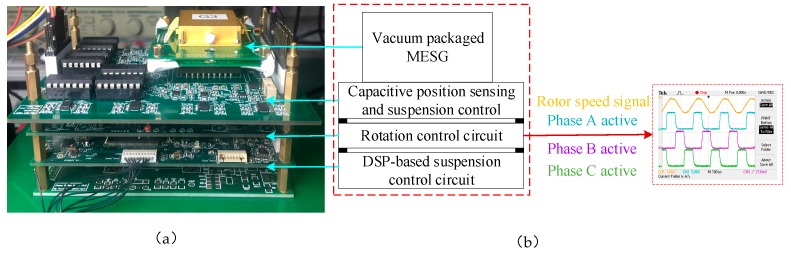
(**a**) Experimental set-up of the MESG and (**b**) the rotor drive waveform.

Figure 9a illustrates an experimental set-up of the MESG prototype comprising three printed circuit boards (PCBs) stacked by customized bus connectors. The MESG chip was mounted on the top PCB along with the capacitive position sensing and associated suspension electronics. The rotation control electronics and the digital suspension controller were realized in the other two PCBs.

A temperature controlled chamber was used to set and maintain constant temperature of the MESG at different settings during temperature drift measurements. A data acquisition system (KEITHLEY 2010 Multimeter, Keithley, Shanghai, China) was used to sample and record the outputs from the MESG and the temperature sensor. Additionally, a control panel programmed by Labview on a laptop functioned as a man-machine interface and used to control the gyroscopic operation and record complete experiment data. 

The rotation of the suspended rotor is based on the principle of a three-phase variable-capacitance electrostatic motor. The 14-pole ring-shaped rotor is misaligned to the 12-pole stator electrodes so that once a drive voltage is applied on the rotation electrodes of one phase, the rotor will be forced to realign to the energized electrodes [16]. To rotate the rotor continuously, the controlled drive voltages are applied with a fixed duty ratio in a phase commutation order of Phases A, B and C, as shown in Figure 9b.

In this section, the experimental results for the scale factor, temperature-induced bias drift, long-term bias stability and temperature drift compensation of the MESG prototype will be presented and discussed.

### 4.2. Scale Factor

Equation (2) indicates that the scale factor is linearly proportional to the rotor speed. To test the scale factor, the MESG was operated at various speed settings of 1.0 × 10^4^, 1.3 × 10^4^ and 1.5 × 10^4^ rpm. The angular rate input was applied by a rate turntable while the MESG output was sampled by the data acquisition system at 1 Hz. 

**Figure 10:**
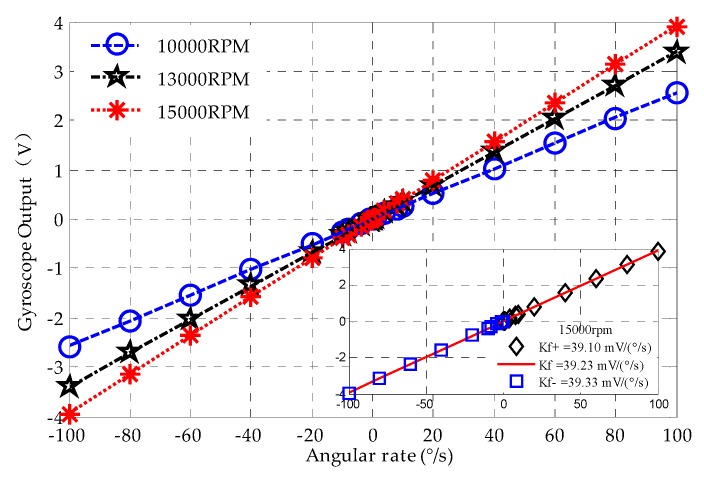
Angular rate input-output characterization of MESG with different rotor speeds.

Figure 10 indicates that the measured scale factor for the MESG operating at different rotor speeds varying from 10,000 rpm to 15,000 rpm. Theoretical and experimental results of the scale factor at different speeds are listed in Table 3 for comparison. It is clear that the experimental results agree well with the theoretical predication by Equation (2). It is interesting to calculate the nonlinearity and asymmetry of the scale factor for performance evaluation of the MESG prototype. Figure 10 shows that the measured nonlinearity and asymmetry at 15,000 rpm are 0.240% and 0.586% with an input range of ± 100°/s, respectively.

### 4.3. Bias Drift

The bias stability is the most crucial index for performance evaluation of such gyroscopes for inertial navigation. The bias drift is highly dependent on temperature variation of the MESG operation [31]. 

#### 4.3.1. Temperature-Dependent Bias Drift

**Figure 11:**
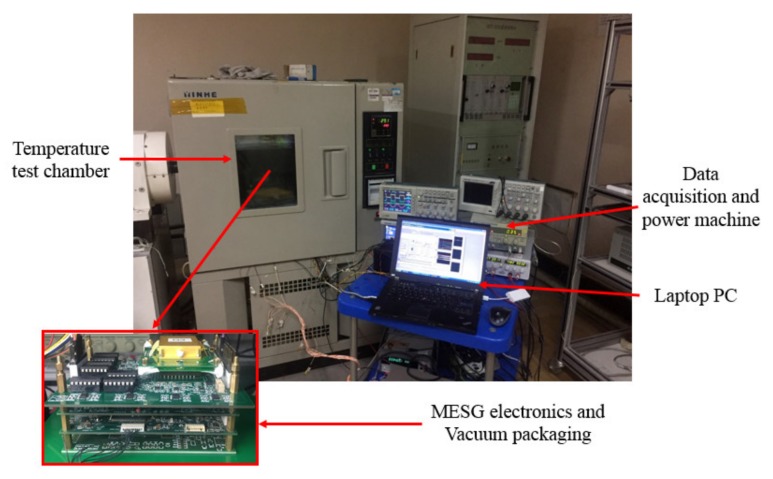
Set-up of the temperature drift testing.

**Figure 12:**
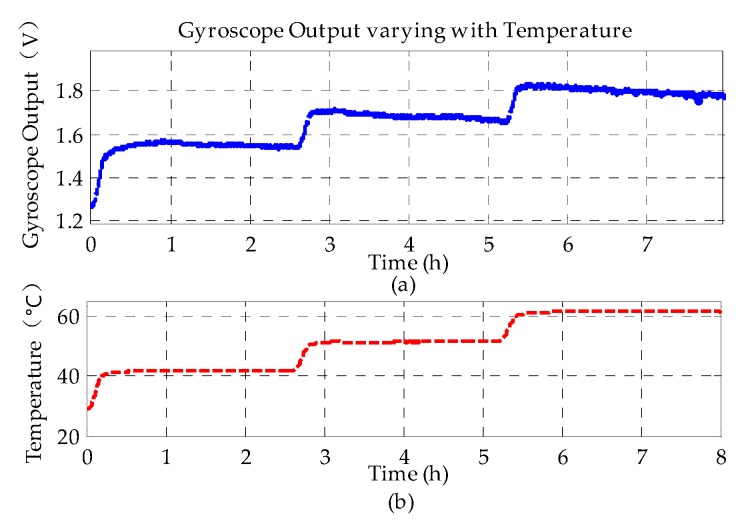
(**a**) Gyroscope output along *h*-axis and (**b**) temperature response varying with time.

To test the temperature-dependent bias drift, the MESG prototype operated at 10,000 rpm was put into the temperature-controlled test chamber with a working temperature ranging from 40 °C to 60 °C, respectively, in 10 °C steps, as shown in Figure 11. A platinum resistance thermometer was embedded inside the MESG package and utilized to sense precisely the real-time temperature of the MESG device. The measurement duration at each temperature point was lasted for nearly 2.5 h to ensure the temperature sensor response would be close to the rotor temperature. Figure 12 shows the bias voltage of the MESG output varies with the working temperature. At each temperature setting, the MESG output increases rapidly as the temperature increases initially; the output voltage decreases much more slowly after the temperature becomes nearly constant.

As can be seen from Figure 12, the angular rate measurement of the MESG is relatively sensitive to variation of the environmental temperature. It was found that the temperature-induced bias drift rate was 12.48 mV/°C (0.49 °s^−1^**/**°C) from the data in Figure 12. 

From the Simulink simulation results in Section 3.4, the temperature drift of gyroscope bias is 8.7 mV/°C. The discrepancy between this experimental results and the simulation results is about 30%, which indicates the temperature drift of the MESG is mainly resulted from thermal stability of the position sensing circuit as well.

#### 4.3.2. Long-Term Bias Stability of the MESG 

To evaluate the long-term bias stability, the MESG prototype was also placed in the temperature-controlled test chamber to maintain a constant temperature environment, which attenuated greatly the drift from ambient temperature variations. 

**Figure 13:**
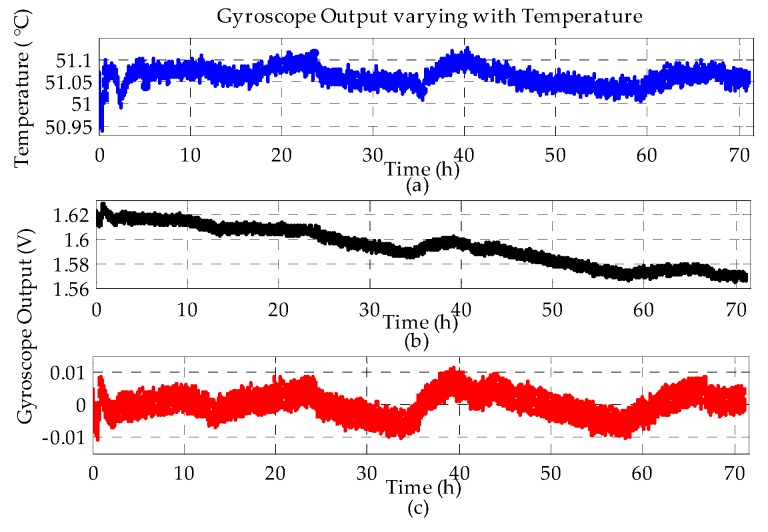
Long-term bias stability in a temperature-controlled environment: (**a**) working temperature; (**b**) raw drift of the MESG bias and (**c**) residual drift compensated by removing the slowly changing time drift.

At a temperature setting of 51 °C, the outputs from the gyroscope and thermometer were sampled by the data acquisition system over 70 h, as shown in Figure 13. 

After the temperature response inside the chamber reaches its steady state, the overall gyroscope output drifts down slowly with time. Considering an extremely small temperature disturbance, as shown in Figure 13a, and the temperature-dependent bias drift in Section 4.3.1, it is clear that the time-dependent drift dominates the long-term bias stability in the temperature controlled environment. Figure 13b indicates that the MESG exhibits a time-dependent bias drift coefficient of 0.737 mV/h, as obtained by least-square fitting the measured data. 

After compensating the time-dependent drift of the MESG output, Figure 13c shows that he residual bias drift is closely correlated with the temperature variation of the MESG. After removing the time drift, the correlation coefficient between the temperature and gyroscope output is 0.6596.

Furthermore, Figure 13b shows that a slowly changing time drift can last over 70 h. Based on the analytical result of the thermal time constant, 35.6 min, given in Section 3.2, it seems that this long-time drift source excludes the possibility of the rotor thermal balance response. Therefore, it is worthwhile to investigate which part of the MESG prototype is the dominant source of the time-dependent bias drift. 

**Figure 14:**
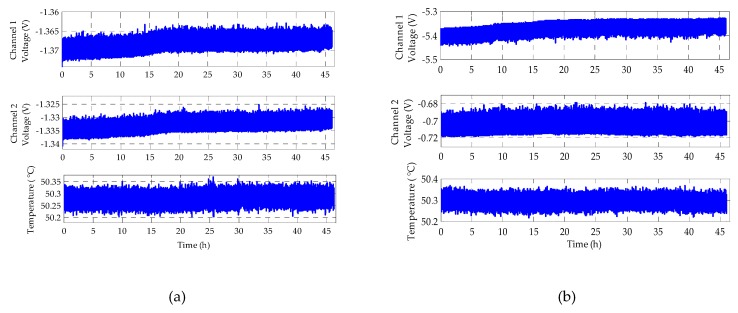
Long-term output drift of position sensing circuit at 50 °C: (**a**) two-channel outputs with similar magnitude and (**b**) two channels with very different outputs.

This time the output time drift of the position sensing channel itself was tested firstly. The position sensing PCB was put into the constant temperature test chamber with a setting of 50 °C and tested for a duration of 46 h. The experimental results of two-channel position sensing output for the *h*-axis measurement are shown in Figure 14.

Figure 14a indicates that the drift rates of Channel 1 and Channel 2 with time are 55.2 and 75.8 μV/h, respectively. Moreover, the simulation result of the rebalance loop indicates that the gyroscope bias drift of the *h* axis generated by the sensing circuit of Channel 1 and Channel 2 in Figure 14a is only −0.642 mV/h. The discrepancy between the experimental drift rate −0.737 mV/h and the simulation result is only 12.89%. It is clear that the long-term bias drift of the MESG is mostly contributed by the position sensing circuit.

To compare the effects of different output voltages on time drift, the amplitude of the position sensing output is adjusted to be larger and smaller relative to its operating voltage, respectively. Figure 14b indicates that the measured drift rates of the two adjusted channels with very different output biases, Channel 1 and Channel 2, are 839 and 36 μV/h, respectively. By comparing the drift rates of these sensing channels in Figure 14a,b, it can be found that the larger of the sensing circuit bias is, the higher the drift rate will be. Therefore, an effective method to reduce the long-term time drift of the MESG bias is to nullify the bias of the position sensing circuit by adjusting the capacitive sensing zero of the suspended rotor.

### 4.4. Compensation of Bias Drift Using a BP Neural Network

The experimental results show that the bias drift of the MESG is both temperature and time dependent. In order to attenuate the drift caused by the temperature and time concurrently, a BP neural network was used to model and compensate for the bias drift of the MESG. Both the temperature and time data were directly fed into the input layer of the BP neural network, while the gyroscope output data were input into the output layer. 

A single hidden layer neural network which includes the input layer, hidden layer and output layer nodes was used in this work. In the MATLAB neural network toolbox, the neural net fitting was used to create the BP neural network. The Levenberg–Marquardt algorithm was chosen for the network training and prediction. In the following compensation experiment, the experimental drift data were divided into a training set (the first 70% of the gyroscopic output data), a validation set (the following 15% of the data) and a test set (the last 15% of the data).

**Figure 15:**
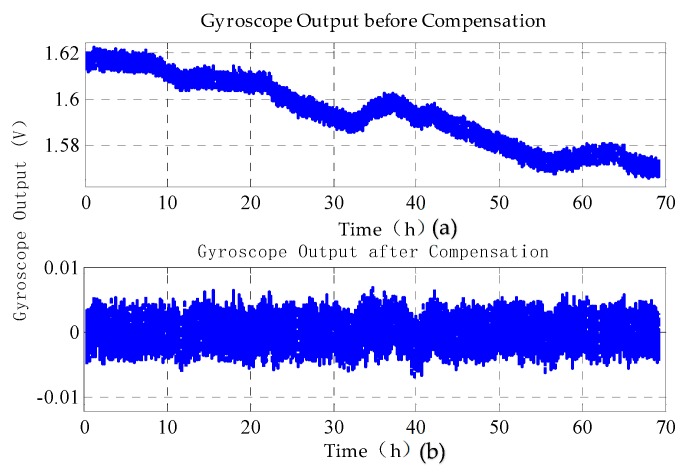
Comparison of the bias drift: (**a**) bias before compensation and (**b**) bias after being compensated by the BP neural network.

After compensation with the BP neural network model, Figure 15 shows that the bias stability of the MESG improves from 0.56 °/s to 0.027 °/s for 70 h and from 0.022 °/s to 0.018 °/s for 1 h data from 23–24 h, respectively. Clearly, the compensation can attenuate the long-term time drift greatly while the short-term 1 h drift is reduced by only 18% due to relatively large noise. In addition, the compensation method is also evaluated in a temperature-uncontrolled environment. The experimental result shows that the bias stability of the MESG improves from 1.2774 °/s to 0.0526 °/s for 24 h. It is clear that the long-term bias drift can be modeled and compensated effectively to improve the MESG performance greatly. 

**Figure 16:**
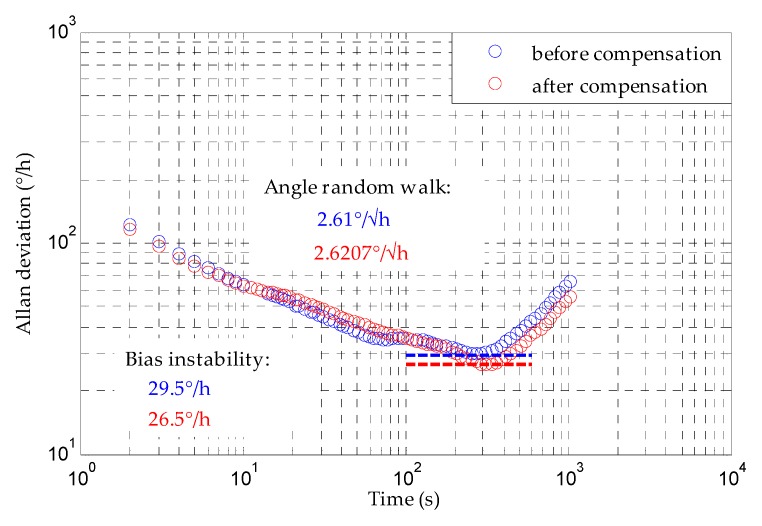
Allan deviation before and after bias compensation.

The Allan deviation analysis with 1-h MESG output data are shown in Figure 16, with and without drift compensation respectively. By comparing these results, it is indicated that the bias instability is improved from 29.5 °/h to 26.5 °/h after compensation. This implies that the compensation can improve the short-term bias performance, but not as much as the long-term result, as discussed above. In addition, the angle random walk is basically the same because it is a representation of the output white noise which can not be reduced by the compensation method. As discussed in Section 2.3, higher vacuum level is an effective solution to reduce the output noise of the MESG. 

## 5. Conclusions

The temperature drift characteristics, long-term drift measurements and compensation of a spinning-rotor MESG have been investigated in this paper. The experimental result indicates that the rotation drive voltage in a form of pulse waveform is a major noise source of the MESG, which can be attenuated by packaging the device under higher vacuum to permit ultra-low drive voltage amplitude. The temperature-dependent characteristics of the rotor structure and capacitive position sensing circuit are analyzed to evaluate the bias drift of the MESG. It was found that the bias drift induced from the position pick-off electronics was virtually four orders of magnitude larger than that from thermal expansion of the rotor structure. The experimental result shows that the MESG bias is relatively sensitive to the temperature variation with a drift rate of 12.48 mV/°C, which is mainly a result of the thermal stability of the position sensing circuit, as predicated theoretically. The long-term bias test results show that both the temperature and time dependent bias drifts exist in the MESG prototype. The long-term bias drift has been modeled and compensated effectively by the BP neural network model to improve significantly the bias stability of the MESG. The future work will focus on reduction of the output noise by packaging the device in higher vacuum and improvement of the long-term stability by optimizing the capacitive position sensing electronics. 

## Figures and Tables

**Table 1:** Main parameters of the micromachined electrostatically suspended gyroscope (MESG) structure.

Description	Value
Rotor outer radius *r*_0_ (μm)	2000
Rotor inner radius *r*_i_ (μm)	1730
Rotor thickness *h* (um)	77
Mass of the rotor *m* (mg)	0.457
Moment of inertia of the rotor *J* (kg·m2)	1.98 × 10^−12^
Axial gap *d*_z_ (μm)	5
Radial gap *d*_r_ (μm)	5
Axial suspension electrode outer radius *R_o_* (μm)	1868
Axial suspension electrode inner radius *R_i_* (μm)	1738
Number of stator phases *N*	3
Number of stator electrodes *N*_s_	24
Number of rotor poles *N*_s1_	14
Angle of rotor poles and stator electrodes *θ*_r_	π/14

**Table 2:** Parameters of the dual-axis MESG rebalance loop.

Description (Unit)	Value
Rotor radial moment of inertia J_e_ (kg·m^2^)	7.86 × 10^−13^
Spin rate of rotor *ɷ*_0_ (rpm)	1.0 × 10^4^
Angular moment of rotor *H* (kg·m^2^·s^−1^)	2.07 × 10^−9^
Torque-voltage coefficient *K*_v_ (N·m·V^−1^)	4.31 × 10^−9^
Angular position stiffness *K*_f_ (N·m·rad^−1^)	2.7 × 10^−5^
Sensitivity of position sensor *K*_s_ (V·rad^−1^)	5.9 × 10^2^

**Table 3:** Comparison between theoretical and experimental scale factors at different rotor speeds.

Rotor Speed	Scale Factor (mV/°/s)			
	Theoretical	Experimental	Nonlinearity	Asymmetry
1.0 × 10^4^ rpm	26.1	25.7	0.236%	1.980%
1.3 × 10^4^ rpm	33.9	33.9	0.120%	0.678%
1.5 × 10^4^ rpm	39.0	39.2	0.240%	0.586%
